# Roles of Cognitive Function on Visual Field Reliability Indices among Glaucoma Patients

**DOI:** 10.3390/jcm12227119

**Published:** 2023-11-15

**Authors:** Aona Ichitani, Eri Takao, Masaki Tanito

**Affiliations:** Department of Ophthalmology, Shimane University Faculty of Medicine, Enya 89-1, Izumo 693-8501, Japan

**Keywords:** mild cognitive impairment, dementia, visual field testing, glaucoma, test reliability

## Abstract

This study reports the prevalence of cognitive impairment (CI) in patients attending a glaucoma outpatient clinic at a tertiary hospital. It also comprehensively assesses possible associations between CI and visual field (VF) reliability indices among glaucoma patients. The retrospective analysis included 1464 eyes from 746 subjects (mean age, 70.6 ± 11.9; 401 males and 345 females). CI was evaluated using the Mini-Cog test, revealing a suspected prevalence of 8.0% (60 out of 746) among the patients. After adjusting for various background parameters using a mixed effects regression model, an abnormal Mini-Cog score was linked to higher false negative (FN) (*p* = 0.0034) and false positive (FP) (*p* = 0.0051) but not fixation loss (FL) (*p* = 0.82). Among the Mini-Cog components, a lower word recall test score was associated with higher FN (*p* < 0.0001), with a borderline difference in FP (*p* = 0.054) and no significant effect on FL (*p* = 0.09). Conversely, a lower clock drawing test score was associated with higher FP (*p* = 0.038), while FL (*p* = 0.49) and FN (*p* = 0.12) remained unaffected. These findings suggest that CI can impact the reliability of VF testing among glaucoma patients, highlighting the importance of assessing cognitive function in glaucoma care.

## 1. Introduction

Glaucoma is a group of ophthalmic neurodegenerative diseases that cause damage to the optic nerve, resulting in visual field (VF) constriction and vision loss [1]. It affects 76 million people worldwide and is projected to increase to 95 million by 2030 [2]. VF testing plays a crucial role in characterizing and monitoring visual loss in glaucoma and ocular hypertension [3]. Among the various methods of VF testing, static perimetry is commonly employed in clinical settings. The global indices, such as mean deviation (MD) and pattern standard deviation (PSD), derived from VF testing using the Humphrey Visual Field Analyzer (Carl Zeiss Meditec, Dublin, CA, USA), are used to estimate the presence and severity of glaucomatous VF defects. To ensure the accuracy of VF testing, reliability indices, such as fixation loss (FL), false-negative (FN), and false-positive (FP) results, are employed. The evaluation of these indices is critical for monitoring glaucoma progression [4,5,6,7,8,9].

Aging is associated with cognitive impairment (CI), which can be attributed to Alzheimer’s disease, vascular dementia, senile dementia, and mild cognitive impairment (MCI). Consequently, glaucoma and CI often coexist. A meta-analysis indicated that the prevalence of glaucoma among individuals with MCI was 7.7%, and the prevalence of dementia ranged from 0.2% to 25.9%. Among people with glaucoma, the prevalence of MCI and dementia ranged from 12.3% to 90.2% and from 2.5% to 3.3%, respectively [10]. In a 3-year population-based prospective cohort study, glaucoma was associated with greater declines in Mental Alternation Test scores and worsening processing speed [11].

Among primary open-angle glaucoma (POAG) cases, worse VF loss was linked to lower scores on the Montreal Cognitive Assessment (MoCA) [12]. CI, as assessed by the telephone version of the MoCA, showed an association between normal tension glaucoma and poor cognitive function [13]. In the population-based Beijing Eye Study, a worse cognitive function score was associated with a higher prevalence of primary angle-closure glaucoma [14]. As a result, the possible association between CI and glaucoma has been reported in various types of glaucoma.

There is a hypothesis suggesting that there is a disease association and shared patho-etiological features between glaucoma and dementia. A hospital-based case–control study indicated that open-angle glaucoma patients may have increased odds of senile dementia, MCI, and other neurodegenerative diseases [15]. In a glaucoma cohort, both functional and structural glaucoma damage were significantly associated with lower cognitive function, independent of age and visual acuity [16]. Postmenopausal women with a large cup-to-disc ratio but without glaucoma or ocular hypertension exhibited lower global cognitive function as assessed by the modified Mini-Mental State Examination (MMSE) [17]. Impairment of cognitive function was observed in patients with POAG who had a thinner lamina cribrosa thickness [18]. Although further research is needed to identify potential causal relationships, glaucoma, Alzheimer’s disease, and Parkinson’s disease share common features in the neuronal damage process [19].

Previously, we reported that aging is associated with decreased reliability in VF testing using a large VF dataset in a real-world setting [20,21]. Each five-point decline in the MoCA score was associated with an increase of 0.23 dB in the prediction error of MD. Cognitive decline was linked to increased VF variability during follow-up [22]. Therefore, the screening and monitoring of CI are crucial in assessing VF progression in the context of glaucoma [22]. Cognitive decline, as assessed by the clock drawing test, was associated with reduced VF reliability, especially with a higher FN rate [23]. Significant negative associations were found between the written MoCA test scores and the FN rate [24]. Thus, age-associated CI can impair individuals’ ability to perform a VF test and compromise the reliability of the results. However, studies focusing on the relationship between CI and VF reliability indices are still limited, and this association requires further elucidation.

The Mini-Cog cognitive function test, a brief cognitive screening test, comprises a three-item word recall and a clock drawing test [25]. The Mini-Cog, scored by an algorithm as “possibly impaired (score ≤ 2)” or “probably normal (score ≥ 3)”, and the Mini-Mental State Examination (MMSE), at a cutpoint of 25, exhibited similar sensitivity (76% vs. 79%) and specificity (89% vs. 88%) for dementia [25]. In this study, the prevalence of CI among patients visiting a glaucoma outpatient clinic at a tertiary hospital was reported. Furthermore, possible associations between CI and VF reliability indices were comprehensively assessed among the glaucoma patients.

## 2. Subjects and Methods

### 2.1. Study Design and Subjects

This retrospective study adhered to the principles of the Declaration of Helsinki, and the Institutional Review Board (IRB) of Shimane University Hospital reviewed and approved the research (study No. 20220616-1, issued on 21 July 2022). IRB approval did not require written informed consent from each patient for publication. Instead, the study protocol was posted at the study institutions to inform participants about the study. This study encompassed 746 subjects (401 males and 345 females), totaling 1464 eyes, who visited the glaucoma outpatient clinic of Shimane University Hospital between March 2020 and April 2022, underwent a cognitive function test with Mini-Cog, and received a VF test with the Central 30-2 program, Humphrey Visual Field Analyzer using the SITA-standard algorithm. Patients with ocular diseases other than glaucoma and cataracts that would cause vision loss were excluded. Therefore, this study included all patients who visited the glaucoma outpatient clinic and met the above criteria and thus included patients with various types of glaucoma as well as those with glaucoma suspects.

### 2.2. Measurements

We retrospectively examined medical records, collecting data on age at Mini-Cog testing, sex, Mini-Cog scores (total score ranging from 0 to 5 points, word recall test score ranging from 0 to 3 points, and clock drawing test score of either 0 or 2 points), best-corrected visual acuity (BCVA), spherical equivalent refractive error (SERE), VF-derived parameters including MD, PSD, and rates of FL, FN, and FP. Our institution routinely administers cognitive function tests to glaucoma clinic patients. Decimal BCVA was converted into the logarithm of the minimum angle of resolution (LogMAR). Decimal visual acuity values of counting fingers, hand motions, light perception, and no light perception were converted to 0.0025, 0.002, 0.0016, and 0.0013, respectively [26]. SERE was measured using autorefractometry (TonoRef III, Nidek, Gamagori, Japan).

### 2.3. Statistical Analysis

The data were presented as mean ± standard deviation (SD) with 95% confidence interval ranges for continuous parameters and in numbers and percentages for categorical parameters. The potential association between normal (≥3) and abnormal (≤2) Mini-Cog scores was evaluated using an unpaired *t*-test for continuous parameters and Fisher’s exact probability test or the Cochran–Armitage trend test for categorical parameters. The potential association between each of the total Mini-Cog scores, word recall test scores, clock drawing test scores, and other parameters was also assessed through one-way ANOVA followed by Tukey–Kramer’s honestly significant difference test. In these assessments, age, sex, and Mini-Cog score were analyzed on a subject-based basis, while VA, SERE, and VF-derived parameters were analyzed on an eye-based basis. These associations were further evaluated through multivariable analysis using a mixed-effects regression model. In the model, eye-based analysis was conducted, and any bias resulting from the inclusion of both eyes from a single subject was adjusted by selecting the subject’s identification number as a random effect. All statistical analyses were performed using JMP Pro statistical software version 16.1.0 (SAS Institute, Inc., Cary, NC, USA). A *p*-value of less than 0.05 was considered statistically significant.

## 3. Results

The demographic characteristics of the patients, including age, sex, Mini-Cog score (total score, word recall test score, clock drawing test score), BCVA, SERE, MD, PSD, FL, FN, and FP, are presented in Table 1. Using a cutoff value of a total Mini-Cog score of ≤2, we found that the suspected prevalence of CI was 8.0% (60 out of 746) among the patients who visited our glaucoma clinic.

Table 2 provides a comparison between groups stratified by the normal/abnormal total Mini-Cog score. In comparison to the normal Mini-Cog score group, the abnormal Mini-Cog score group exhibited several significant differences. These differences included older age (*p* < 0.0001), worse word recall (*p* < 0.0001) and clock drawing (*p* < 0.0001) test scores, worse BCVA (*p* = 0.0001), less myopic SERE (*p* < 0.0001), and worse MD (*p* = 0.038). However, sex and PSD remained equivalent between the two groups. Regarding VF reliability indices, the abnormal Mini-Cog score group had significantly higher FN (*p* < 0.0001) and FP (*p* = 0.011) rates compared to the normal Mini-Cog score group, while the FL rate (*p* = 0.59) was equivalent between the groups.

Table 3 presents the univariate analysis of various parameters with the total Mini-Cog score. A lower Mini-Cog score was associated with worse BCVA (*p* < 0.0001), less myopic SERE (*p* < 0.0001), higher FL (*p* = 0.037), and increased FN (*p* < 0.0001). However, there was no significant correlation with MD, PSD, or FP. The post hoc test revealed a significant difference in Mini-Cog score between scores of 0 and 5 in FL, as well as between any pair of scores of 0, 2, 3, and 4 in FN. 

Table 4 shows the results of univariate analysis between word recall test scores and various parameters. Worse BCVA (*p* = 0.0006), less myopic SERE (*p* < 0.0001), higher FL (*p* = 0.023), and higher FN (*p* < 0.0001) were correlated with lower word recall test scores, while MD, PSD, and FP were not significantly correlated. Post hoc tests revealed significant differences in word recall test scores between scores of 1 and 3 in FL and between any pair of scores of 0, 1, 2, and 3 in FN.

Table 5 presents the results of univariate analysis between clock drawing test scores and various parameters. When comparing the group with higher clock drawing test scores (score = 2) to the group with worse scores (score = 0), the worse score group exhibited the following: worse BCVA (*p* < 0.0001), less myopic SERE (*p* = 0.011), higher PSD (*p* = 0.031), higher FN (*p* < 0.0001), and higher FP (*p* = 0.0022). However, there were no significant differences in MD and FL between the two score groups.

**Figure 1:**
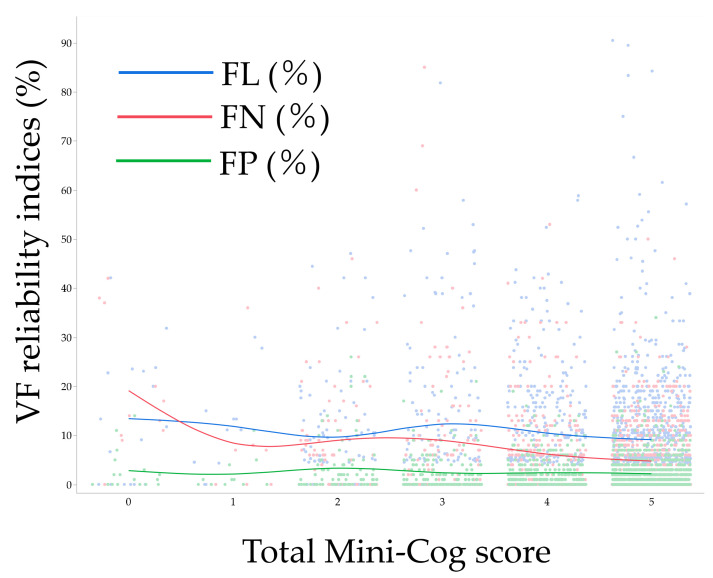
Association between total Mini-Cog score and visual field reliability indices. Each line indicates a moving average. VF, visual field; FL, fixation loss; FN, false negative; FP, false positive.

Figure 1 illustrates the distribution of each VF reliability index in association with the total Mini-Cog score, revealing distinct trends in the distribution of each reliability index. Table 6 displays the results of the multivariable analysis model (Model 1) examining factors associated with VF reliability indices. Following adjustment for various background parameters, an abnormal Mini-Cog score was linked to higher FN (*p* = 0.0034) and FP (*p* = 0.0051) but not with FL (*p* = 0.82). Additionally, aging was associated with higher FL (*p* = 0.0011) and FN (*p* = 0.023). Females exhibited a higher association with FN (*p* = 0.038) and FP (*p* = 0.029) compared to males, while higher MD was correlated with increased FN (*p* < 0.0001) and FP (*p* < 0.0001). Lastly, higher PSD was associated with higher FP (*p* < 0.0001).

**Figure 2:**
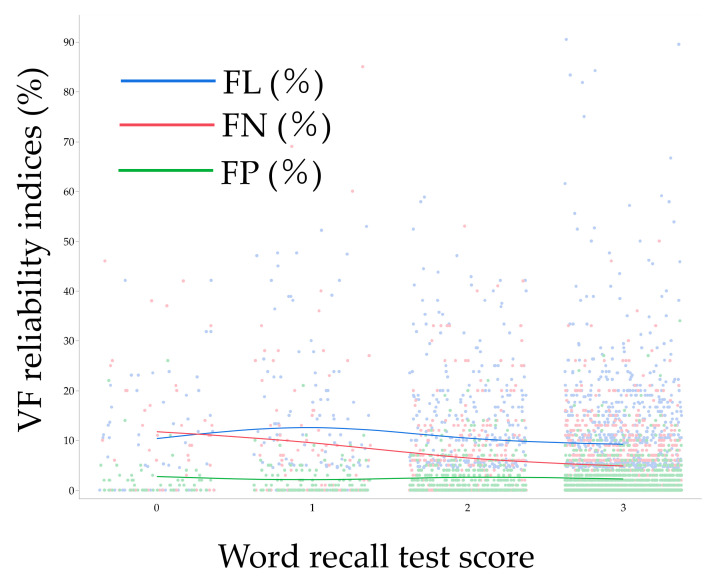
Association between word recall test score and visual field reliability indices. Each line indicates a moving average. VF, visual field; FL, fixation loss; FN, false negative; FP, false positive.

Figure 2 shows the distribution of each VF reliability index in association with word recall test score. Table 7 shows the multivariable analysis model (i.e., Model 2) for factors associated with VF reliability indices. After adjustment for various background parameters, a lower word recall test score was associated with higher FN (*p* < 0.0001), while the difference was borderline with FP (*p* = 0.054) and not with FL (*p* = 0.09). Among the pairs of each score group, FN was different between scores of 1 and 2 (*p* = 0.016), and FP was different between scores of 2 and 3 (*p* = 0.043). Aging was also associated with higher FL (*p* = 0.0090). Females were associated with higher FL (*p* = 0.045), FN (*p* = 0.011), and FP (*p* = 0.054) than males; higher MD was associated with higher FN (*p* < 0.0001) and FP (*p* < 0.0001); and higher PSD was associated with higher FP (*p* < 0.0001).

**Figure 3:**
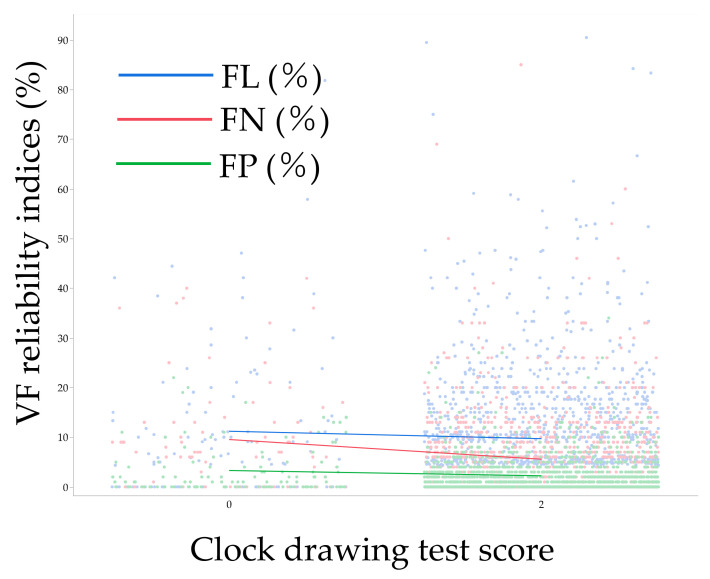
Association between clock drawing test score and visual field reliability indices. Each line indicates a moving average. VF, visual field; FL, fixation loss; FN, false negative; FP, false positive.

Figure 3 shows the distribution of each VF reliability index in association with clock drawing test score. Table 8 shows a multivariable analysis model (i.e., Model 3) for factors associated with VF reliability indices. After adjustment for various background parameters, a lower clock drawing test score was associated with higher FP (*p* = 0.038), while not with FL (*p* = 0.49) and FN (*p* = 0.12). Aging was also associated with higher FL (*p* = 0.0010) and FN (*p* = 0.0077). Females were associated with higher FL (*p* = 0.087), FN (*p* = 0.043), and FP (*p* = 0.036) than males; higher MD was associated with higher FN (*p* < 0.0001) and FP (*p* < 0.0001); and higher PSD was associated with higher FP (*p* < 0.0001).

## 4. Discussion

This study aimed to investigate the potential impact of CI on the reliability of VF testing in real-world settings among glaucoma patients. Our findings indicate that 8% of the patients visiting the glaucoma clinic were suspected of having CI, as determined by the Mini-Cog test. Through both univariate and multivariable analyses, we observed that the Mini-Cog score was associated with VF reliability indices. However, the nature of this association varied between the two different components of the Mini-Cog score. Previous research has explored the relationship between CI and VF reliability in smaller patient samples using different cognitive assessment tools, such as the MMSE (51 patients) [27], MoCA test (61 patients) [24], and clock drawing test (60 patients) [23]. To our knowledge, this is the first study to investigate this relationship using the Mini-Cog test, and it involves the largest dataset, comprising 1464 eyes from 746 subjects.

In a meta-analysis that considered various CI screening methods, the prevalence of MCI among glaucoma patients ranged from 12.3% to 90.2%, while the prevalence of dementia ranged from 2.5% to 3.3% [10]. Therefore, our observed prevalence of CI may appear lower compared to previous reports. The choice of the Mini-Cog score cutoff of ≤2 for identifying CI provided similar sensitivity and specificity for dementia as the MMSE at a cutoff of 25 [25]. When we applied a cutoff of Mini-Cog score ≤3, the calculated prevalence of CI was 17.8% (133 out of 746). This figure aligns more closely with previous reports, suggesting that the choice of screening test may contribute to the observed differences. MCI represents an intermediate stage between normal aging and dementia, with dementia itself encompassing various stages from mild to advanced [28]. CI screening tests like the Mini-Cog are typically not suitable for advanced dementia cases, as they primarily target MCI and mild-to-moderate dementia. Similarly, VF testing is not feasible in advanced dementia patients. Therefore, we expect substantial overlap between Mini-Cog-eligible and VF test-eligible patients.

Our analysis revealed differences in the total Mini-Cog score between FN and FP when the score was categorized (Table 2), but differences were observed in FL and FP when the score was treated as an ordinal variable in univariate analysis (Table 3). Previous studies found that MMSE-based CI was not significantly associated with FL, FN, and FP [27]. In contrast, MoCA-based CI was associated with FN without affecting FL and FP [24]. Using the clock drawing test, all three indices—FL, FN, and FP—were associated with CI in univariate analysis, while only FN was associated with CI in multivariable analysis [23]. Given that aging is the direct and strongest factor influencing cognitive function, other than the screening methods for CI, differences in patients’ backgrounds, such as age and study settings, can thus be associated with the discrepancy. The effects of SERE observed in univariate analyses (Table 2 and Table 3) disappeared in multivariable analysis (Table 6), and this can be attributed to covariate effects related to aging. Prior studies have reported the impact of aging on VF sensitivity [16,20,21], which aligns with our observation of a significant association between CI and MD (Table 2). In multivariable analysis, we also noted higher FL, FN, and FP in females compared to males (Table 6). The reasons for this gender difference remain unknown and require further investigation.

When considering each component of the Mini-Cog score, multivariable analysis revealed a significant association between FN and word recall test score (Table 7), while FP was exclusively associated with the clock drawing test (Table 8). This unique observation suggests that each component of the Mini-Cog score has varying effects on different VF reliability indices, hinting at the presence of underlying mechanisms or reasons for these differences. MCI can be broadly categorized into two subtypes: amnestic MCI, which resembles an early stage of Alzheimer’s disease (AD), and non-amnestic MCI, encompassing more heterogeneous types of dementia, including vascular dementia, frontotemporal dementia, or dementia with Lewy bodies [28]. Amnestic MCI is characterized by memory impairment, while non-amnestic MCI exhibits other cognitive dysfunctions, such as executive impairments, attention disorders, and visuospatial cognitive impairments [28]. This speculation, while inconclusive in this study, suggests that FN may be more affected by memory dysfunctions, represented by the word recall test, while FP may be more influenced by non-memory cognitive dysfunctions, such as executive and visuospatial cognitive impairments, represented by the clock drawing test. Further research is required to confirm these hypotheses.

This study has several limitations. Like other retrospective studies, it may be subject to patient selection bias. The study site served as a tertiary care center to which many patients were referred for surgery or additional therapy due to inadequate reduction in intraocular pressure (IOP) after initial treatment. Additionally, this study was conducted in an area of Japan with a notably aged population. The study subjects comprised various types of glaucoma and some glaucoma suspects. Although difficult to perform in this study, future studies should compare cognitive function and visual field reliability across disease types. Factors like fatigue and loss of concentration, which could influence reliability indices, were not assessed. Previous studies have reported that longer testing times were associated with wider variations in reliability indices [29,30,31,32]. The absence of information regarding ocular pathology and clinical backgrounds, such as visual acuity and IOP, is another limitation. Consequently, it is possible that variations in VF reliability indices stem from ocular pathologies and physical factors. Despite these limitations, we believe that this study’s results reflect real-world outcomes influenced by diverse pathological and background factors. More specifically, our findings provide important insights into the relationship between glaucoma, visual field testing, and cognitive function.

## 5. Conclusions

This study identified that 8% of patients visiting the glaucoma clinic were suspected to have CI, as determined by the Mini-Cog test. Through multivariable analyses, it was observed that a total Mini-Cog score of ≤2 was associated with higher FN and FP. Additionally, lower scores on the word recall test and the clock drawing test were associated with higher FN and FP, respectively. These findings suggest that CI can impact the reliability of VF testing among glaucoma patients. Early detection and assessment of CI, as provided by tools like the Mini-Cog, could aid in the interpretation of VF results and improve the overall management of glaucoma patients.

## Figures and Tables

**Table 1:** Demographic Data Based on Subjects and Eyes.

Parameters	N or Mean ± SD	% or 95% CI Range
Subjects	746	
Age, years	70.6 ± 11.9	69.8, 71.5
Sex		
Male	401	54
Female	345	46
Mini-Cog score		
0	10	1
1	6	1
2	44	6
3	73	10
4	163	22
5	450	60
Word recall		
0	30	4
1	59	8
2	187	25
3	470	63
Clock drawing		
0	60	8
2	686	92
Eyes	1464	
BCVA, LogMAR	0.21 ± 0.45	0.19, 0.24
SERE, D	−2.1 ± 3.2	−2.3, −1.9
MD, dB	−7.9 ± 6.0	−8.3, −7.6
PSD, dB	8.2 ± 4.6	7.9, 8.4
FL, %	9.8 ± 12.0	9.2, 10.5
FN, %	5.8 ± 8.2	5.4, 6.2
FP, %	2.3 ± 3.5	2.1, 2.5

SD, standard deviation; CI, confidence interval; BCVA, best-corrected visual acuity; LogMAR, logarithm of minimal angle of resolution; SERE, spherical equivalent refractive error; D, diopter; MD, mean deviation; PSD, pattern standard deviation; dB, decibel; FL, fixation loss; FN, false negative; FP, false positive.

**Table 2:** Comparison between groups stratified by Mini-Cog score.

Parameters	Mini-Cog Score, ≤2		Mini-Cog Score, ≥3		*p*-Value
	N or Mean ± SD	% or 95% CI Range	N or Mean ± SD	% or 95% CI Range	
Subjects	60	8	686	92	
Age, years	69.9 ± 11.6	69.3, 70.5	80.5 ± 7.5	79.1, 81.9	<0.0001
Sex					
Male	29	48	372	54	0.42
Female	31	52	314	46	
Word recall					
0	30	50	0	0	<0.0001
1	6	10	53	8	
2	24	40	163	24	
3	0	0	470	69	
Clock drawing					
0	40	67	20	3	<0.0001
2	20	33	666	97	
Eyes	114	8	1350	92	
BCVA, LogMAR	0.37 ± 0.67	0.25, 0.49	0.20 ± 0.43	0.18, 0.22	0.0001
SERE, D	−0.68 ± 1.5	−1.0, −0.39	−2.2 ± 3.3	−2.4, −2.1	<0.0001
MD, dB	−9.5 ± 6.0	−11.0, −7.9	−7.9 ± 6.0	−8.2, −7.5	0.038
PSD, dB	7.6 ± 3.9	6.9, 8.3	8.2 ± 4.6	8.0, 8.5	0.15
FL, %	10.4 ± 11.5	8.3, 12.6	9.8 ± 12.1	9.1, 10.4	0.59
FN, %	10.3 ± 11.5	7.8, 12.8	5.5 ± 7.9	5.1, 5.9	<0.0001
FP, %	3.1 ± 5.1	2.2, 4.1	2.3 ± 3.3	2.1, 2.4	0.011

*p*-values are calculated by unpaired *t*-test for continuous data and by Fisher’s exact probability test or Cochran–Armitage trend test for categorical data. SD, standard deviation; CI, confidence interval; BCVA, best-corrected visual acuity; LogMAR, logarithm of minimal angle of resolution; SERE, spherical equivalent refractive error; D, diopter; MD, mean deviation; PSD, pattern standard deviation; dB, decibel; FL, fixation loss; FN, false negative; FP, false positive.

**Table 3:** Univariate analysis between total Mini-Cog score and various parameters.

Parameters	Mini-Cog Score						*p*-Value †
	0	1	2	3	4	5	
BCVA, LogMAR	0.34 ± 0.48	0.64 ± 0.86	0.34 ± 0.62	0.32 ± 0.53	0.21 ± 0.44	0.18 ± 0.41	<0.0001 **
*p*-value ‡					vs. 1 *	vs. 1 **, 2 *, 3 **	
SERE, D	−0.12 ± 0.75	−1.0 ± 1.8	−0.75 ± 1.6	−1.75 ± 3.0	−1.7 ± 3.4	−2.5 ± 3.3	<0.0001 **
*p*-value ‡						vs. 0 *, 2 **, 4 **	
MD, dB	−10.0 ± 6.2	−5.5 ± 2.9	−9.8 ± 6.1	−8.1 ± 6.0	−7.7 ± 6.0	−7.9 ± 5.9	0.22
PSD, dB	5.9 ± 2.7	7.3 ± 3.1	7.9 ± 4.1	7.8 ± 4.2	7.9 ± 4.5	8.4 ± 4.7	0.14
FL, %	13.4 ± 13.1	11.9 ± 9.9	9.6 ± 11.3	12.3 ± 15.4	10.5 ± 11.5	9.1 ± 11.6	0.037 *
*p*-value ‡						vs. 3 *	
FN, %	19.2 ± 13.7	8.3 ± 12.8	9.0 ± 10.5	9.0 ± 13.7	6.2 ± 8.4	4.8 ± 6.3	<0.0001 **
*p*-value ‡			vs. 0 **	vs. 0 **	vs. 0 **, 3 *	vs. 0 **, 2 **, 3 **	
FP, %	2.8 ± 4.2	2.1 ± 3.4	3.3 ± 5.5	2.4 ± 3.6	2.4 ± 3.2	2.2 ± 3.3	0.12

Data are expressed as mean ± standard deviation. *p*-values are calculated by one-way ANOVA (†) followed by Tukey–Kramer’s honestly significant difference test (‡). * and ** indicate *p* < 0.05 and *p* < 0.01, respectively. BCVA, best-corrected visual acuity; LogMAR, logarithm of minimal angle of resolution; SERE, spherical equivalent refractive error; D, diopter; MD, mean deviation; PSD, pattern standard deviation; dB, decibel; FL, fixation loss; FN, false negative; FP, false positive.

**Table 4:** Univariate analysis between word recall test score and various parameters.

Parameters	Word Recall Test Score				*p*-Value †
	0	1	2	3	
BCVA, LogMAR	0.32 ± 0.61	0.34 ± 0.59	0.23 ± 0.46	0.18 ± 0.41	0.0006 **
*p*-value ‡				vs. 1 **	
SERE, D	−0.65 ± 1.3	−1.3 ± 2.6	−1.6 ± 3.2	−2.5 ± 3.3	<0.0001 **
*p*-value ‡				vs. 0 **, 1 **, 2 **	
MD, dB	−9.2 ± 6.0	−8.0 ± 5.7	−8.0 ± 6.1	−7.9 ± 6.0	0.66
PSD, dB	7.6 ± 4.1	7.9 ± 4.0	7.9 ± 4.4	8.3 ± 4.7	0.26
FL, %	10.4 ± 11.1	12.6 ± 14.0	10.4 ± 11.6	9.2 ± 12.0	0.023 *
*p*-value ‡				vs. 1 *	
FN, %	11.7 ± 12.6	9.5 ± 14.8	6.5 ± 8.6	4.8 ± 6.4	<0.0001 **
*p*-value ‡			vs 0 **,1 **	vs. 0 **, 1 **, 2 *	
FP, %	2.7 ± 5.0	2.1 ± 3.2	2.6 ± 3.6	2.2 ± 3.4	0.27

Data are expressed as mean ± standard deviation. *p*-values are calculated by one-way ANOVA (†) followed by Tukey–Kramer’s honestly significant difference test (‡). * and ** indicate *p* < 0.05 and *p* < 0.01, respectively. BCVA, best-corrected visual acuity; LogMAR, logarithm of minimal angle of resolution; SERE, spherical equivalent refractive error; D, diopter; MD, mean deviation; PSD, pattern standard deviation; dB, decibel; FL, fixation loss; FN, false negative; FP, false positive.

**Table 5:** Univariate analysis between clock drawing test score and various parameters.

Parameters	Clock Drawing Test Score		*p*-Value
	0	2	
BCVA, LogMAR	0.38 ± 0.56	0.20 ± 0.44	<0.0001 **
SERE, D	−1.4 ± 2.6	−2.2 ± 3.3	0.011 *
MD, dB	−8.9 ± 6.2	−7.9 ± 5.9	0.18
PSD, dB	7.3 ± 3.9	8.2 ± 4.6	0.031 *
FL, %	11.2 ± 14.3	9.7 ± 11.8	0.21
FN, %	9.6 ± 10.8	5.6 ± 8.0	<0.0001 **
FP, %	3.3 ± 4.9	2.2 ± 3.4	0.0022 **

Data are expressed as mean ± standard deviation. *p*-values are calculated by unpaired *t*-tests. * and ** indicate *p* < 0.05 and *p* < 0.01, respectively. BCVA, best-corrected visual acuity; LogMAR, logarithm of minimal angle of resolution; SERE, spherical equivalent refractive error; D, diopter; MD, mean deviation; PSD, pattern standard deviation; dB, decibel; FL, fixation loss; FN, false negative; FP, false positive.

**Table 6:** Multivariable analysis for factors associated with visual field reliability indices, Model 1.

Parameters	FL(%)			FN(%)			FP(%)		
	Estimate	95% CI	*p*-Value	Estimate	95% CI	*p*-Value	Estimate	95% CI	*p*-Value
Age, year	0.13	0.052, 0.20	0.0011 **	0.049	0.007, 0.091	0.023 *	0.008	−0.014, 0.031	0.47
Sex, F/M	0.72	−0.080, 1.52	0.078	0.46	0.025, 0.90	0.038 *	0.27	0.028, 0.51	0.029 *
Mini-Cog, ≤2/≥3	0.41	−3.04, 3.85	0.82	2.85	0.95, 4.76	0.0034 **	1.48	0.45, 2.51	0.0051 **
BCVA, LogMAR	1.80	−0.72, 4.32	0.16	−0.36	−1.85, 1.13	0.64	−0.28	−1.04, 0.48	0.47
SERE, D	−0.029	−0.28, 0.23	0.82	0.024	−0.12, 0.17	0.74	−0.043	−0.12, 0.034	0.28
MD, dB	0.16	−0.066, 0.38	0.17	−0.34	−0.47, −0.22	<0.0001 **	0.18	0.11, 0.25	<0.0001 **
PSD, dB	−0.068	−0.34, 0.20	0.62	−0.13	−0.29, 0.029	0.11	0.18	0.098, 0.26	<0.0001 **

*p*-values are calculated by mixed effects regression model. * and ** indicate *p* < 0.05 and *p* < 0.01, respectively. BCVA, best-corrected visual acuity; LogMAR, logarithm of minimal angle of resolution; SERE, spherical equivalent refractive error; D, diopter; MD, mean deviation; PSD, pattern standard deviation; dB, decibel; FL, fixation loss; FN, false negative; FP, false positive.

**Table 7:** Multivariable analysis for factors associated with visual field reliability indices, Model 2.

Parameters	FL(%)			FN(%)			FP(%)		
	Estimate	95% CI	*p*-Value	Estimate	95% CI	*p*-Value	Estimate	95% CI	*p*-Value
Age, year	0.10	0.026, 0.18	0.0090 **	0.035	−0.008, 0.078	0.11	0.006	−0.017, 0.030	0.59
Sex, F/M	0.82	0.017, 1.62	0.045 *	0.57	0.13, 1.01	0.011 *	0.30	0.054, 0.54	0.017 *
Word recall test score	-	-	0.09	-	-	<0.0001 **	-	-	0.054
2–3	1.1	−0.83, 3.04	0.26	0.50	−0.55, 1.55	0.35	0.60	0.018, 1.18	0.043 *
1–2	2.97	−0.49, 6.43	0.093	2.35	0.45, 4.25	0.016 *	−0.35	−1.39, 0.69	0.51
0–1	−2.67	−8.27, 2.93	0.35	2.36	−0.73, 5.45	0.13	1.38	−0.31, 3.07	0.11
BCVA, LogMAR	1.78	−0.74, 4.30	0.17	−0.34	−1.83, 1.15	0.65	−0.25	−1.02, 0.51	0.52
SERE, D	−0.042	−0.30, 0.21	0.74	0.022	−0.12, 0.16	0.76	−0.042	−0.12, 0.035	0.28
MD, dB	0.16	−0.058, 0.38	0.15	−0.34	−0.47, −0.21	<0.0001 **	0.18	0.11, 0.25	<0.0001 **
PSD, dB	−0.062	−0.33, 0.21	0.65	−0.13	−0.29, 0.029	0.11	0.18	0.10, 0.26	<0.0001 **

*p*-values are calculated by mixed effects regression model. * and ** indicate *p* < 0.05 and *p* < 0.01, respectively. BCVA, best-corrected visual acuity; LogMAR, logarithm of minimal angle of resolution; SERE, spherical equivalent refractive error; D, diopter; MD, mean deviation; PSD, pattern standard deviation; dB, decibel; FL, fixation loss; FN, false negative; FP, false positive.

**Table 8:** Multivariable analysis for factors associated with visual field reliability indices, Model 3.

Parameters	FL(%)			FN(%)			FP(%)		
	Estimate	95% CI	*p*-Value	Estimate	95% CI	*p*-Value	Estimate	95% CI	*p*-Value
Age, year	0.13	0.051, 0.20	0.0010 **	0.057	0.015, 0.099	0.0077 **	0.012	−0.011, 0.035	0.30
Sex, F/M	0.70	−0.10, 1.51	0.087	0.46	0.015, 0.90	0.043 *	0.26	0.017, 0.50	0.036 *
Clock drawing test, 0/2	1.21	−2.22, 4.65	0.49	1.50	−0.40, 3.40	0.12	1.09	0.061, 2.13	0.038 *
BCVA, LogMAR	1.79	−0.73, 4.30	0.16	−0.44	−1.93, 1.06	0.57	−0.32	−1.08, 0.45	0.41
SERE, D	−0.029	−0.28, 0.23	0.82	0.031	−0.11, 0.17	0.67	−0.039	−0.12, 0.038	0.32
MD, dB	0.16	−0.06, 0.38	0.16	−0.35	−0.48, −0.22	<0.0001 **	0.18	0.11, 0.24	<0.0001 **
PSD, dB	−0.065	−0.34, 0.21	0.64	−0.13	−0.29, 0.025	0.10	0.18	0.096, 0.26	<0.0001 **

*p*-values are calculated by mixed effects regression model. * and ** indicate *p* < 0.05 and *p* < 0.01, respectively. BCVA, best-corrected visual acuity; LogMAR, logarithm of minimal angle of resolution; SERE, spherical equivalent refractive error; D, diopter; MD, mean deviation; PSD, pattern standard deviation; dB, decibel; FL, fixation loss; FN, false negative; FP, false positive.

## Data Availability

Data are fully available upon reasonable request to the corresponding author.
